# Assessing the effects of group perinatal compassion focused therapy in a National Health Service Talking Therapies service in England

**DOI:** 10.3389/fpsyg.2026.1800377

**Published:** 2026-03-26

**Authors:** Leah A Millard-Brewer, Anja Wittkowski

**Affiliations:** 1 Division of Psychology and Mental Health, School of Health Sciences, The University of Manchester, Manchester, United Kingdom; 2 Perinatal Mental Health and Parenting Research Unit, Greater Manchester Mental Health NHS Foundation Trust, Manchester, United Kingdom; 3 Manchester Academic Health Science Centre, The University of Manchester, Manchester, United Kingdom

**Keywords:** mothers, parent, parent-infant, psychological therapy, self-compassion, women

## Abstract

**Background:**

To address the prevalence of perinatal mental health difficulties in the United Kingdom (UK), the National Health Service’s (NHS) Talking Therapies for anxiety and depression programme aims to prioritise perinatal service users to receive evidence-based psychological therapy. Perinatal compassion focused therapy (P-CFT) is an intervention being offered to perinatal women as part of this aim. This evaluation intends to provide a preliminary exploration of P-CFT’s acceptability and potential benefits to service users.

**Methods:**

In this repeated measures cohort design, a retrospective analysis using data from P-CFT groups delivered between 2021 and 2024 on service users’ levels of mood (anxiety and depression), compassion (self-criticism and self-reassurance), postpartum bonding, and group attendance were examined through analyses between pre- and postintervention scores.

**Results:**

Thirty-six perinatal women were included in the evaluation in ten P-CFT groups. Using paired samples t-tests (*n* = 36), the findings indicated that P-CFT led to significant improvements in compassion alongside reductions in mood-related symptoms and any self-reported difficulties in postpartum bonding. Effect sizes ranged from moderate to high. The average retention rate across the included ten groups was 48.0%.

**Conclusion:**

The results indicated that completion of the P-CFT groups might potentially benefit mothers in the perinatal period. Nevertheless, the overall low retention rate suggested potentially poor acceptability of the intervention or poor engagement related to their ability to attend, or other feasibility factors. Further investigation is required to explore the potential facilitators and barriers to implementing P-CFT in perinatal mental health settings, including NHS Talking Therapies settings.

## 1 Introduction

In the perinatal period, which ranges from conception up to two years postpartum, anxiety and/or depression carries a global prevalence of affecting approximately one in five women (Howard and Khalifeh, 2020; The Lancet, 2023). Without intervention, perinatal mental health difficulties can significantly affect the wellbeing of the mother.^1^ For instance, mothers are at high risk of a new or recurring mental health difficulty during the early postpartum period, with one to two in every 1,000 births requiring a psychiatric inpatient admission (Howard and Khalifeh, 2020). Furthermore, mental health-related difficulties are the leading cause of maternal deaths (from six weeks to one-year postpartum) in the United Kingdom (MBRRACE-UK, 2025). In addition, perinatal mental health difficulties can negatively affect the parent-infant relationship. Such a disruption may elicit the adverse development of a child’s cognitive, social, and emotional abilities (Erickson et al., 2019). Therefore, due to the time-sensitive nature of the perinatal period, it is important for mothers to have timely access to evidence-based psychological interventions to effectively treat perinatal mental health difficulties (O’Mahen et al., 2023).

To improve the delivery of and accessibility to evidence-based psychological therapies in the United Kingdom (UK), the National Health Service (NHS) Talking Therapies for anxiety and depression (previously referred to as Improving Access to Psychological Therapies, IAPT) was developed (NHS Talking Therapies, 2024). Approximately, 1.2 million people were able to access an NHS Talking Therapies for anxiety and depression service from the year 2021 to 2022 (NHS Talking Therapies, 2024). It is recommended that Talking Therapies services prioritise the perinatal population to receive psychotherapeutic treatment within an appropriate timeframe (O’Mahen et al., 2023). As O’Mahen et al. (2023) reported, many expectant and postpartum women were eligible to access this service to treat perinatal anxiety and/or depression.

One therapy offered by NHS England’s Talking Therapy services to address perinatal mental health problems is Cree’s (2010, 2015) perinatal compassion focused therapy (i.e., P-CFT). Delivered typically across 12 weekly sessions, the therapy is an adaptation of Gilbert’s (2009, 2014) original compassion focused therapy (CFT) model of 10–12 sessions. From theories of evolutionary psychology to Buddhist philosophies, compassion-focused therapy (CFT; Gilbert, 2009, 2014) is a transdiagnostic therapy underpinned by a diverse range of psychological approaches. A primary theory of Gilbert’s (2009, 2014) compassion-focused model is that mental health difficulties are underpinned by negative thought patterns, such as shame, self-criticism and guilt (Gilbert and Irons, 2004). Therefore, CFT aims to substitute negatively affiliated thought and behavioural patterns with more positively affiliated patterns like warmth, contentment and self-compassion. CFT theorises that such patterns are rooted across three emotional regulatory systems: the threat system (attributed to fight-or-flight), the drive system (attributed to reward and excitement), and the soothing-oxytocin system (attributed to self-compassion and self-warmth; Gilbert, 2009, 2014). According to CFT, higher levels of shame and self-criticism are associated with an overactive threat system, which consequently supresses the soothing-oxytocin system. Therefore, thought and behavioural patterns characterised by self-compassion (which is required to counterbalance shame and self-criticism) is consequently inaccessible. As part of CFT, individuals are taught the required compassion-based knowledge and skills to facilitate the re-activation of the soothing-oxytocin system (Gilbert, 2009, 2014).

A mother’s level of self-criticism increases through pregnancy to her postpartum period (Brassel et al., 2019). Some researchers have attributed this increase in self-criticism to society’s unrealistic expectations of motherhood that mothers evaluate themselves against, which can elicit feelings of shame and self-inadequacy (Raneberg and MacCallum, 2025; Sonnenburg and Miller, 2021). As well as affecting a mother’s mental wellbeing, this increase of self-criticism may also mediate the quality of the parent-infant bond (Beato et al., 2022). Thus, higher levels of maternal self-criticism may disrupt the relationship between mother and infant. P-CFT aims to address the exacerbation in feelings of shame and guilt that are often prevalent during pregnancy and the postpartum period, with an additional focus on the parent-infant relationship (Cree, 2010).

Meta-analyses of CFT have indicated its potential benefits for individuals with mental health difficulties in terms of reductions in clinical symptoms and improvements in self-compassion (Kirby et al., 2017; Millard et al., 2023; Petrocchi et al., 2024). Furthermore, Garrett et al.’s (2025) recent qualitative synthesis of 12 studies reported CFT to have high levels of acceptability across a diverse range of clinical groups. However, none of these reviews included enough studies studies that could help inform the potential benefits of CFT/P-CFT for the perinatal population.

In their mini-review, Millard and Wittkowski (2023) identified only five studies from 2018 to 2023 (*n* = 1,258) that evaluated either Gilbert’s (2009, 2014) model of CFT or Cree’s (2010, 2015) P-CFT adaptation in mothers from baseline to post-intervention. Despite reporting significant improvements in compassion-based outcomes, four of the five studies were derived from non-clinical samples. Therefore, the review provided only preliminary insights into the benefits of CFT/P-CFT for mothers experiencing sub-clinical mental health symptoms.

Since Millard and Wittkowski’s (2023) review, Lawrence et al. (2024) analysed routinely collected measures from women (*n* = 114) who participated in online P-CFT groups within a specialist perinatal community mental health teams (P-CMHT) in the Northwest of England from 2020 to 2023. Women were offered ten weekly sessions based on Cree’s (2015) P-CFT adaptation. Using paired samples t-tests, Lawrence et al. (2024) compared scores on the *Forms of Self-Criticising/Attacking and Self-reassurance* scale (*FSCRS*; Gilbert et al., 2004), the *Postpartum Bonding Questionnaire* (*PBQ*, Brockington et al., 2006) and the *Clinical Outcomes in Routine Evaluaton-10* (*CORE-10*; Barkham et al., 2013). In this service evaluation, the authors reported statistically significant improvements in postpartum bonding and self-reassurance and significant reductions in levels of self-criticism and psychological distress with medium to large effect sizes. Similar findings were reported by Thirkettle et al. (2024) in a sample of 34 women in a specialist perinatal CMHT in the East of England. In addition, Raneberg and MacCallum’s (2025) cross-sectional study of 499 mothers in the UK explored the role of shame and self-compassion in maternal ambivalence (the presence of a mother’s concurrent positive and negative feelings towards her role as a mother and towards her children). In their online survey, the authors found that maternal ambivalence was associated with higher self-reported scores of depression and anxiety, which were mediated through stronger shame and lower levels of self-compassion (Raneberg and MacCallum, 2025). Therefore, these studies further emphasise the potential importance of mothers receiving compassion-focused interventions to treat perinatal mental health difficulties.

Although service evaluations such as Lawrence et al. (2024) and Thirkettle et al. (2024) offer an insight into the potential benefits of P-CFT in specialist P-CMHTs, it is important to also collect feasibility data to inform the implementation of perinatal interventions in other mental health services, such as the Talking Therapies service. Thus, this study aimed to examine the feasibility of offering P-CFT to perinatal women in a Talking Therapies service, and the acceptability of the intervention’s content, delivery and format through the exploration of group attendance and retention rates. Secondly, we aimed to explore potential change in relevant outcomes from baseline to post-intervention.

## 2 Method

### 2.1 Design and study setting

Adopting a repeated measures cohort design, routinely collected data across two NHS Talking Therapies teams in Northwest England were analysed. Ten P-CFT groups were delivered from 2021 to 2024. The study was part of a larger project on P-CFT led by the first author (Millard-Brewer et al., 2025). Approval for the larger project was granted by the NHS National Research Ethics Service via the London - Camden and Kings Cross Research Ethics Committee (REC reference number: 23/LO/0383) in May 2023, with an amendment to include this current study being granted in November 2024.

### 2.2 Participant inclusion and exclusion criteria

All mothers were required to meet service criteria to be referred into the Talking Therapies service. Following referral to the Talking Therapies service, the service users were screened for Suitability for the P-CFT group. As the data were routinely collected by staff, the current study adopted the following inclusion criteria: Mothers who attended a P-CFT group in either Site 1 or Site 2 between 2021 and 2024 and completed at least one routine outcome measure at baseline and post-intervention. Regarding exclusion criteria, the therapy was delivered in English only. Therefore, non-English speakers were not invited by the service to attend the group because provisions for a translator were not available.

### 2.3 Intervention and recruitment procedure

An overview of the service’s adaptation of Cree’s (2010, 2015) model of P-CFT is outlined in Table 1. Typically, groups were comprised of either eight or 12 sessions (see Supplementary materials). Over time, group facilitators in the service adapted the P-CFT groups, leading to a reduction in the number of sessions offered. Each two-hour session was facilitated by two qualified therapists, with at least one facilitator having accreditation in CFT/P-CFT. Groups were delivered either remotely via an online conferencing software (e.g., Microsoft Teams) or in-person (see Supplementary materials for details).

**Table 1:** Overview of P-CFT sessions based on Michelle Cree’s model of P-CFT.

Session	Theme	Content	At-home exercises
1	Introduction to compassion	Introduction to the three emotion regulatory systems (drive, threat and soothing-oxytocin)	Reflect on one’s own three systems
2	Developing the soothing system	Soothing rhythm breathing; old brain vs. new brain; ‘tricky brain’; mindfulness exercises	Practise soothing rhythm breathing
3	The three systems in action and self-criticism	The role of the three systems in real-life scenarios; Introduction to self-criticism	Mindfulness
4	Self-criticism and compassionate Imagery	Safe place imagery; critical inner voice; critical teacher versus compassionate teacher	Safe place imagery
5	Understanding ourselves and compassionate flow	Attachment system and the three emotion regulatory systems; three flows of compassion; compassionate-self-imagery exercises; compassionate attributes	Compassionate Imagery for each flow of compassion
6	Blocks to compassion	Overcoming objections to self-compassion	Reading materials on how to manage fears, blocks and resistances to compassion
7	Our compassionate nurturer and compassionate thinking	Creating an ideal compassionate nurturer and an ideal compassionate image; How to generate compassionate thoughts	The Compassionate Nurturer; Compassionate Thought record
8	Our emotions and future wellbeing	Recognising emotions; Compassionate postcard; summary and reflections of learnings	Continuing with techniques learnt in a manageable way

Women accessed the Talking Therapies service either via self-referral or their General Practitioner (GP). Typically, service users are offered an initial screening assessment with a therapist (in-person or online), when they are asked to complete the *Generalised Anxiety Disorder-7* (*GAD-7*; Spitzer et al., 2006) and the *Public Health Questionnaire-9* (*PHQ-9*; Kroenke et al., 1999) and assess their current needs (see Table 2). If suitable, service users were then referred to the P-CFT group or an alternative psychological intervention. Questionnaires were completed remotely via an online link prior to each assessment and therapy session.

**Table 2:** List of self-reported outcome measures completed at pre- and post-intervention.

Outcome measure	Aim	Number of items	Scoring range	Scoring	Scale type	Response descriptions	Psychometric properties	
							Internal reliability (Cronbach’s alpha)	Test retest reliability
*GAD-7* total score	Assesses severity of anxiety symptoms	7	0–21	Higher score indicative of increased severity	4-point Likert scale	‘Not at all’ to ‘Nearly every day’	0.89 (Ayers et al., 2024)	ICC = 0.83 (Simpson et al., 2014)
*PHQ-9* total score	Detects severity of depressive symptoms	9	0-27	Higher score indicative of increased severity	4-point Likert scale	‘Not at all’ to ‘Nearly every day’	0.80 (Cheng et al., 2025)	*r* = 0.75 (Wang et al., 2021)
*PBQ* total score	Measures difficulties in the mother-infant bond	25	0–125	General bonding difficulties, cut off score ≥ 26. Severe bonding difficulties, cut off score ≥ 40	6-point Likert scale	‘Always’ to ‘Never’	0.63–0.79 (Mathews et al., 2019)	ICC = 0.78 (Torres-Giménez et al., 2021)
*FSCRS* subscales: *Hated self (HS)* *Inadequate self (IS)* *Reassured self (RS)*	Assessing levels of self-criticism and self-reassurance	22	0–36 0–20 0–32	Higher score indicative of increased self-criticism (*HS, IS*) and reassured self (*RS*)	5-point Likert scale	‘Not at all like me’ to ‘Extremely like me’	0.81–0.91 (Castilho et al., 2015)	*r* = 0.65–0.72 (Castilho et al., 2015)

ICC, Intraclass correlation coefficient; Generalised Anxiety Disorder-7 (GAD-7; Spitzer et al., 2006); Public Health Questionnaire (PBQ-9; Kroenke et al., 1999); Postpartum Bonding Questionnaire (PBQ; Brockington et al., 2006); Fears of Self-Criticism/Self-Attacking and Self-Reassurance (FSCRS; Gilbert et al., 2004).

### 2.4 Outcome measures

Service users were asked to complete four outcome measures (see Table 2). The measures were collected at pre- and post-intervention. The following demographic information was also collected: Gender, age at the time of the first P-CFT session, ethnicity, occupation, parity, primary health diagnosis, and age of infant at the time of the first P-CFT session.

During the first session of P-CFT, group participants were asked to complete the *Forms of Self-Criticising/Attacking and Self-Reassuring* scale (*FSCRS*; Gilbert et al., 2004) and the *Postpartum Bonding Questionnaire* (*PBQ*, Brockington et al., 2006) measures (see Table 2). Following the final session of the P-CFT group (8, 10 or 12 weeks later), service users were asked to complete the previous outcome measures again. Mothers were either then discharged from the Talking Therapies service or referred on for further psychological treatment.

### 2.5 Statistical analysis

All analyses were conducted using IBM SPSS Statistics (IBM CORP, Version 29.0). Demographic data and the self-reported outcome measures (*PHQ-9*, *GAD-7*, *FSCRS* subscales, *PBQ*) at baseline and post-intervention were presented using summary statistics (means, standard deviations or percentages). Shapiro–Wilk tests for normality were conducted (see Supplementary materials).

To explore the feasibility and acceptability of P-CFT, percentages of attrition and retention rates were analysed (the difference in the number of attendees between the first and last session of each group). To measure potential changes in scores from baseline to post-intervention, the *GAD-7*, *FSCRS* and *PBQ* were analysed using paired samples t-tests. Mean differences, standard deviation (SD), 95% confidence intervals, and Cohen’s *d* to determine effect size were also reported. Non-parametric data (*PHQ-9*) were analysed using the Wilcoxon Signed-Rank test. For effect size of the *PHQ-9*, the *r* statistic was reported. Furthermore, individual scores were analysed using Jacobson and Truax’s (1991) reliable change index (RCI) for each measure to assess the effects of P-CFT at an individual level. Using a score of > 1.96 to signify improvement, the number of participants to reach this threshold were calculated. Participant data were only analysed if outcome measures were completed at both timepoints.

## 3 Results

Across three years from 2021 to 2024, ten P-CFT groups were delivered in total. Participant data were derived from six groups at Site 1 and four groups at Site 2 involving 36 women. Table 3 displays a summary of participant demographic and other information. The average age of the participants was 34 (SD = 6.04), 64% identified as White British, and the average parity was 1.4. Data were normally distributed (see Supplementary materials), except for the PHQ-9 baseline scores.

**Table 3:** Demographic and psychiatric characteristics of participants and infants.

Characteristic	Detail	Total (*n* = 36)
Gender, % (*n*)	Woman	100% (36)
Mean age, years (SD)		34 (6.04)
Ethnicity, % (*n*)	White British	63.9% (23)
	White Irish	2.8% (1)
	Asian or Asian British	5.6% (2)
	Black or Black British	2.8% (1)
	Mixed White and Black African	2.8% (1)
	Mixed White and Black Caribbean	2.8% (1)
	Mixed White and Asian	2.8% (1)
	Other	2.8% (1)
	Not stated	5.6% (2)
Occupation, % (*n*)	Homemaker	25.0% (9)
	Full-time employment	19.4% (7)
	Part-time employment	2.8% (1)
	No employment/Receiving state support	5.6% (2)
	Not stated	47.2% (17)
Parity, *M* (SD)		1.4 (0.9)
Primary mental health diagnosis, % (*n*)	Adjustment disorder	58.3% (21)
	Mixed anxiety and depression	36.1% (13)
	Depressive episode	2.8% (1)
	Posttraumatic stress disorder (PTSD)	2.8% (1)
Infant characteristics	Age range	0–2 years

### 3.1 Feasibility and acceptability indicators

Due to the low retention rate of one group at Site 2 (14.3%), the group was terminated prematurely, and no participant outcome data were available. However, the group was included in the following retention analysis. Retention rates for each group ranged from 14.3 to 100%, with an overall average retention rate of 48.0% across all ten groups that were offered. Site 1 and Site 2 obtained average retention rates of 48.5 and 47.6%, respectively across their P-CFT groups. The low retention rates of the P-CFT groups indicates that the results of this study are preliminary and must be viewed with substantial caution.

#### 3.1.1 Mood

For depression, the Wilcoxon signed ranked test revealed that the baseline scores of the PHQ-9 were significantly lower at post-intervention following the P-CFT group; *z* = −3.76, *p* = <0.001 (see Table 4 for details). Similarly, scores from the paired samples t-test of *GAD-7* revealed a significant reduction in self-reported levels of anxiety from baseline to post-intervention; *t* (34) = 5.40, *p* = <0.001. As displayed in Tables 4, 5, medium to large effect sizes were observed. The RCI indicated that 48.6% and 42.9% of women reported clinically meaningful change in the *PHQ-9* and the *GAD-7*, respectively.

**Table 4:** Wilcoxon signed-rank test.

Outcome measure	Baseline score Mdn	Post-intervention score Mdn	*z*	*p*	*r*
*PHQ-9*	12.00	7.00	−3.76	<0.001	0.64

**Table 5:** Paired samples test and effect sizes.

Outcome measure	Baseline scores	Post-intervention scores	Paired mean difference (SD)	95% confidence interval of the difference		Paired *t* test		
	M (*SD*)	M (SD)		Lower	Upper	*t*	*p*	Cohen’s *d*
*GAD-7*	11.17 (4.29)	7.69 (5.28)	3.49 (3.82)	2.17	4.80	5.40	<0.001	0.91
*FSCRS Inadequate self*	24.55 (7.25)	17.61 (6.52)	6.94 (8.56)	3.80	10.07	4.51	<0.001	0.81
*FSCRS Hated self*	6.81 (5.21)	4.19 (3.54)	2.61 (5.08)	0.748	4.48	2.86	0.008	0.79
*FSCRS Reassured self*	13.39 (6.33)	17.58 (6.51)	−4.19 (7.29)	−6.87	−1.52	4.70	<0.001	−0.56
*PBQ*	20.52 (10.57)	12.65 (7.12)	7.87 (8.02)	4.40	11.34	4.70	<0.001	0.98

#### 3.1.2 Self-criticism

Further paired samples t-tests revealed that baseline scores of the *FSCRS Inadequate self* subscales reduced at post-intervention highlighting a statistically significant reduction in levels of self-criticism; *t* (30) = 4.51, *p* = <0.001. Levels of self-criticism were also observed from the *FSCRS Hated self* subscale at baseline versus post-intervention; *t* (30) = 2.86, *p* = 0.008. Improvements in self-reassurance were also observed following a significant increase in the scores of the *FSCRS Reassured self* subscale from baseline (*M =* 13.39, SD = 6.33) and post-intervention (*M* = 17.58, SD = 6.51), *t* (22) = 4.70, *p* = <0.001. Across the three subscales, effect sizes ranged from medium to large (see Table 5). The RCI revealed 48.4% (*FSCRS Inadequate self*), 22.6% (*FSCRS Hated self*), and 38.7% (*FSCRS Reassured self*) of the service users reached clinically significant change.

#### 3.1.3 Postpartum bonding

Twenty-three of the 36 participants completed the postpartum bonding measures (*PBQ*) at both timepoints. Significant reductions in perceived difficulties in bonding were observed from the start of the P-CFT group to the final P-CFT session; *t* (22) = 4.70, *p* = <0.001. Clinically significant change was observed in 56.2% of the service users. However, it is important to note that the average PBQ baseline score (*M* = 20.52, SD = 10.57) did not reach the PBQ cut-off score for general parent-infant bonding difficulties (≥ 26). Twenty-six percent (*n* = 6) reported a score above the PBQ threshold for general parent-infant bonding difficulties and 4.3% (*n* = 1) scored above the severe bonding difficulties cut-off score (≥ 40).

## 4 Discussion

Through analysing routinely collected measures, this study aimed to conduct a preliminary exploration into the acceptability and feasibility of P-CFT in an NHS Talking Therapies service. Specifically, this study sought to examine changes in self-reported measures of mood, levels of compassion and postpartum bonding. Findings of this study indicated that P-CFT was beneficial for perinatal service users who were experiencing mild to moderate mental health difficulties in relation to the included outcome measures. Effect sizes were predominantly in the moderate to high range. Nevertheless, across each measure the percentage of service users that observed clinically meaningful change ranged from 38.7% to 56.2%. Given that datasets from only 36 perinatal women were available for analysis, despite a timeframe of three years, means that these results are preliminary and must be viewed with strong caution.

In relation to Lawrence et al. (2024) and Thirkettle et al. (2024) who evaluated P-CFT for more complex mental health difficulties in specialist perinatal mental health services, this study provided similar significant findings in that they observed significant reductions in self-criticism and improvements in self-reassurance using the *FSCRS* subscales. This study also reflected Lawrence et al.’s (2024) significant reductions in difficulties in postpartum bonding through the *PBQ* outcome measure.

### 4.1 Strengths and limitations

Although the results indicated benefits to P-CFT, this study was restricted by its low methodological rigour. Particularly, the data could not determine if the effects of the P-CFT group were maintained over a longer period of time. To acquire a deeper understanding of the effects of P-CFT on mothers and their infants, prospective studies using both quantitative and qualitative methods are required.

Due to the two services adapting the P-CFT group over time, any direct comparisons between each of the P-CFT groups in this evaluation were limited. This heterogeneity in group format further accentuates the preliminary nature of these findings and limits their generalisability to other P-CFT groups in NHS Talking Therapies programmes in the UK, and other perinatal-related mental health services.

### 4.2 Clinical implications

The potential benefits of P-CFT indicated that the intervention had positive clinical implications for perinatal women. However, the low retention rates may have indicated poor acceptability for P-CFT in the Talking Therapies service. Groups retained an average 48.0% of service users from baseline to post-intervention. Comparatively, in Lawrence et al.’s (2024) evaluation of specialist perinatal services for women with moderate to severe perinatal mental health difficulties, 75.4% percent of service users (*n* = 86) attended all ten P-CFT online group sessions. In a similar service to the latter, 73% of participants (*n* = 25) completed six to eight sessions of their online eight-session P-CFT programme (Thirkettle et al., 2024). The reasoning behind the higher number of attrition rates between this current study and Lawrence et al.’s (2024) and Thirkettle et al.’s (2024) evaluations are unclear. Dropout rates were perhaps caused by low acceptability of the service’s delivery.

However, the attrition rates might reflect the feasibility of P-CFT in a specific clinical service, rather than the acceptability of the intervention itself. Talking Therapies services are commonly associated with high levels of staff turnover and burnout (Steel et al., 2015). One implication associated with staff wellbeing could be low retention rates. Thus, the drop-out rates reported in this study might reflect a universal issue of retention across NHS Talking Therapies programmes. A Freedom of Information (FOI) request reported 45% of referrals from the general population to Talking Therapies services between 2022 and 2023 did not complete treatment, and 29.1% attended only one session (Scott, 2024). Other explanations for attrition might be due to the local service’s missed appointment procedures. For instance, one IAPT/Talking Therapies service adopted a policy of immediate discharge following two consecutive non-attendances (Binnie and Boden, 2016), which is reflected in other Talking Therapies services.

The foregoing statistics of Talking Therapies programmes might explain the hindered retention rates of the interventions embedded in the service, such as P-CFT. For example, in their meta-synthesis of CFT groups across 12 studies, Garrett et al. (2025) proposed several clinical recommendations for the delivery of this intervention. To retain service users, group facilitators may need to provide additional support to service users due to the challenging nature of compassion-focused exercises (Garrett et al., 2025). Practically, this additional support required for P-CFT may not be feasible in a service such as Talking Therapies due to the aforementioned factors like high staff turnover and burnout. However, this study cannot determine if such factors were applicable in this current study. Nevertheless, the significant improvement in outcomes reported in this study infer that P-CFT was beneficial for the completers of the P-CFT groups.

As shown in the Supplementary materials, 11 of the 12 groups were delivered online. With the high attrition rates, one must consider if the mode of delivery was a contributing factor to the high dropout rates. In their randomised controlled trial of 910 perinatal women with postpartum depression who received an online behavioural-activation treatment, O'Mahen et al. (2013) reported high attrition rates, despite their intervention’s effectiveness in reducing levels of depression. Furthermore, Li et al.’s (2023) systematic review of 13 studies (*n* = 2,158 perinatal women) that explored the effectiveness of online psychological interventions for perinatal mental health difficulties reported high rates of satisfaction across the studies. However, dropout rates ranged from 2.6% to 60.8% across those 13 studies. Factors such as acceptability and duration of the intervention and the client-therapist relationship must be acknowledged when aiming to reduce attrition rates (Li et al., 2023). Based on the high attrition rates of this current study, it is possible that the mothers struggled with the length of the intervention (ranging from eight to 12 weeks). Moreover, 13 mothers who took part in a perinatal psychodynamic group therapy for anxiety and depression expressed that the online aspect created a sense of disconnectedness from the therapy (Pollack et al., 2024). Factors such as the presence of a child in the home during the remote delivery of the intervention may also understandably lead to difficulties in a mother’s ability to engage. Unfortunately, information on how many people declined to attend the therapy based on its delivery format were not available for analysis. For any future trial, the collection of such information at the point of referral to the P-CFT group would be advisable to inform the acceptability of the therapy format.

Furthermore, low retention rates in the current study might have been explained by women experiencing beneficial changes after only attending a limited number of sessions. Therefore, women might have not wished to complete the whole three-month course of P-CFT. However, the delivery of session-by-session outcome measures by the therapy facilitators would be warranted to investigate the reasons of retention rates further.

In addition, this study has provided insight into the selection of outcome measures in NHS mental health services. For the two evaluations of P-CFT that were conducted in P-CMHTs, the selected measure for mood-related symptoms differed. Lawrence et al. (2024) and Thirkettle et al. (2024) administered the *CORE-10*, and the *CORE-34* (Evans et al., 2002), respectively. The *PHQ-9* and the *GAD-7* were administered to measure mood-related symptoms in this current study, in contrast Thirkettle et al. (2024) also included an additional compassion-related measure, the *Fears of Compassions scale* (Gilbert et al., 2011). Heterogeneity in outcome measures was observed in Millard et al.’s (2023) systematic review and meta-analysis of CFT in clinical populations and is a common occurrence in mental health research (Veal et al., 2024). A lack of standardisation across clinical services restricts future comparisons for any prospective evaluations of psychological interventions, such as P-CFT.

### 4.3 Future directions

Despite the fact that the Talking Therapies service runs across a large geographical area in an ethnically diverse city, the applicability of the findings is primarily limited to White British mothers. It is unclear why the data were derived from mostly White mothers. However, in a qualitative study, 18 mothers, who self-identified as being from an ethnic minority, reported experiencing additional barriers to accessing perinatal mental health care, such as family and community beliefs with regard to mental health care, inter-generational trauma and lack of cultural awareness from healthcare providers (Pilav et al., 2022). Therefore, further investigation is required to explore if P-CFT is both accessible and beneficial to mothers from ethnically minoritised backgrounds.

An investigation into the acceptability and feasibility of P-CFT groups in mental health services with stronger methodological rigour is warranted, such as adopting a mixed methods approach. Nevertheless, a mixed methods feasibility study is currently being conducted with P-CFT groups in P-CMHTs for service users who experience severe and/or complex mental health difficulties (Millard-Brewer et al., 2025). However, a further understanding of the benefits of P-CFT in parents who have milder to moderate presentations of mental health difficulties is still warranted.

## 5 Conclusion

For perinatal mothers, this current study has indicated that completing a P-CFT group in an NHS Talking Therapies programme may significantly improve levels of mood and postpartum bonding and improve levels of compassion. However, these preliminary findings must be interpreted with extreme caution. Undermined by the low retention rates, the acceptability and feasibility of P-CFT groups in NHS Talking Therapies services are still undetermined. Further exploration of this intervention within this specific service is required before conclusions can be drawn.

## Data Availability

The raw data supporting the conclusions of this article will be made available by the authors, upon reasonable request.

## Footnotes

1 In line with Darwin et al. (2021), for this paper the term ‘mother’ also includes trans men and non-binary individuals.
