# Correction: Assessing the effects of group perinatal compassion focused therapy in a National Health Service Talking Therapies service in England

**DOI:** 10.3389/fpsyg.2026.1914053

**Published:** 2026-07-22

**Authors:** Leah A. Millard-Brewer, Anja Wittkowski

**Affiliations:** 1 Division of Psychology and Mental Health, School of Health Sciences, The University of Manchester, Manchester, United Kingdom; 2 Perinatal Mental Health and Parenting Research Unit, Greater Manchester Mental Health NHS Foundation Trust, Manchester, United Kingdom; 3 Manchester Academic Health Science Centre, The University of Manchester, Manchester, United Kingdom

**Keywords:** mothers, parent, parent-infant, psychological therapy, self-compassion, women

In the published article, there was a mistake in Table 2. In the fourth column “Scoring Range”, the PHQ-9 total score is incorrectly stated as being “0–21”. The corrected the PHQ-9 total score should be read as “0–27”.

The correct Table 2 appears below.

**Table 2:** List of self-reported outcome measures completed at pre- and post-intervention.

Outcome measure	Aim	Number of items	Scoring range	Scoring	Scale type	Response descriptions	Psychometric properties	
							Internal reliability (Cronbach's alpha)	Test retest reliability
*GAD-7* total score	Assesses severity of anxiety symptoms	7	0–21	Higher score indicative of increased severity	4-point Likert scale	‘Not at all' to ‘Nearly every day'	0.89 (Ayers et al. 2024)	ICC = 0.83 (Simpson et al., 2014)
*PHQ-9* total score	Detects severity of depressive symptoms	9	0–27	Higher score indicative of increased severity	4-point Likert scale	‘Not at all' to ‘Nearly every day'	0.80 (Cheng et al., 2025)	*r* = 0.75 (Wang et al. 2021)
*PBQ* total score	Measures difficulties in the mother-infant bond	25	0–125	General bonding difficulties, cut off score ≥ 26. Severe bonding difficulties, cut off score ≥ 40	6-point Likert scale	‘Always' to ‘Never'	0.63–0.79 (Matthews et al., 2019)	ICC = 0.78 (Torres-Giménez et al., 2021)
*FSCRS* subscales: *Hated self (HS*) *Inadequate self (IS)* *Reassured self (RS)*	Assessing levels of self-criticism and self-reassurance	22	0–36 0–20 0–32	Higher score indicative of increased self-criticism (*HS, IS*) and reassured self (*RS*)	5-point Likert scale	‘Not at all like me' to ‘Extremely like me'	0.81–0.91 (Castilho et al., 2015)	*r* = 0.65–0.72 (Castilho et al., 2015)

ICC, Intraclass correlation coefficient; Generalised Anxiety Disorder-7 (GAD-7; Spitzer et al., 2006); Public Health Questionnaire (PBQ-9; Kroenke et al., 1999); Postpartum Bonding Questionnaire (PBQ; Brockington et al., 2006); Fears of Self-Criticism/Self-Attacking and Self-Reassurance (FSCRS; Gilbert et al., 2004).

The **Acknowledgments** statement is incorrect as published. The incorrect statement read as “We are grateful to the Perinatal Champions within the Greater Manchester Mental Health NHS Foundation Trust's Talking Therapies services who assisted with the data extraction for this evaluation. Please also note that the first author Leah Millard-Brewer has previously published research under the name, Leah Millard.”

The corrected statement should now read as: “We are grateful to the Perinatal Champions within the Greater Manchester Mental Health NHS Foundation Trust's Talking Therapies services who assisted with the data extraction for this evaluation and facilitated the P-CFT groups. The Perinatal Champions were Amy Yates (North Manchester Team; CBT Therapist), Kathryn Mylona (North Manchester Team; CBT Therapist), Dimitra Ioannou (South Manchester Team; CBT Therapist), Danielle Sedgewick (South Manchester Team; CBT Therapist) and Hannah Thomas (South Manchester Team; Counsellor). Please also note that the first author Leah Millard-Brewer has previously published research under the name, Leah Millard.”

The original version of this article has been updated.

