# Supplementary material for: Assessing the effects of group perinatal compassion focused therapy in a National Health Service Talking Therapies service in England
Source: Front Psychol. 2026 Mar 26;17:1800377. doi: 10.3389/fpsyg.2026.1800377 (PMC13062174; doi:10.3389/fpsyg.2026.1800377)
Supplement: Supplementary file 1 [file Data_Sheet_1.PDF]

Overview of P-CFT delivery and attendance in chronological order across Site 1 and Site 2

Group	Site	Number of sessions	Mode of delivery	First session attendance (*n*)	Final session attendance (*n*)
1	1	12	In-person	3	3
2	1	*	Online	7	1
3	2	12	Online	9	5
4	2	12	Online	10	4
5	1	10	Online	11	4
6	2	12	Online	8	3
7	1	8	Online	8	6
8	2	8	Online	6	4
9	1	8	Online	9	4
10	1	8	Online	4	2

**Group ended prematurely due to high dropout rate*

Shapiro-Wilk tests for normality

The following variables reached levels of normality: *GAD-7* (Pre-test scores W = .97, p = .709 ; post-test W = .93, p = .097), *FSCRS Inadequate Self subscale* (Pre-test scores W = .95, p = .366; post-test W = .95, p = .274), *FSCRS Hated Self* subscale (Pre-test scores W = .95, p = .266 ; post-test W = .92, p = .071), *FSCRS Reassured Self* (Pre-test scores W = .97, p = .748; post-test W = .95, p = .354), and *PBQ* total score (Pre-test scores W = .94, p = .244; post-test W = .94, p = .188). In addition, visual examinations of the QQ plots were conducted, which determined the decision to use parametric tests. The *PHQ-9* post-intervention scores did not reach levels of normality (Pre-test scores W = .98, p = .815; post-test W = .82, p = <.001); therefore, the non-parametric test was selected. The skewness and kurtosis values for the data were .491 and .932, respectively.
